# Acute dose-dependent effects and self-guided titration of continuous *N,N-*dimethyltryptamine infusions in a double-blind placebo-controlled study in healthy participants

**DOI:** 10.1038/s41386-024-02041-8

**Published:** 2024-12-19

**Authors:** Livio Erne, Severin B. Vogt, Lorenz Müller, Albiona Nuraj, Anna Becker, Aaron Klaiber, Melani Zuparic, Nimmy Varghese, Anne Eckert, Deborah Rudin, Dino Luethi, Matthias E. Liechti

**Affiliations:** 1 Clinical Pharmacology and Toxicology, Department of Biomedicine and Department of Clinical Research, University Hospital Basel, Basel, Switzerland; 2 Department of Pharmaceutical Sciences, University of Basel, Basel, Switzerland; 3 Psychiatric University Hospital, University of Basel, Basel, Switzerland; 4 Transfaculty Research Platform Molecular and Cognitive Neuroscience, University of Basel, Basel, Switzerland

**Keywords:** Drug development, Translational research

## Abstract

*N,N*-dimethyltryptamine (DMT) is a serotonergic psychedelic that is known for its short-lasting effects when administered intravenously. Several studies have investigated the administration of intravenous boluses or combinations of a bolus and a subsequent continuous infusion. However, data on dose-dependent acute effects and pharmacokinetics of continuous DMT infusions are lacking. We used a double-blind, randomized, placebo-controlled, crossover design in 22 healthy participants (11 women, 11 men) who received placebo and DMT (0.6, 1.2, 1.8, and 2.4 mg/min) over an infusion duration of 120 min. We also tested a self-guided titration scheme that allowed participants to adjust the DMT dose rate at prespecified time points to achieve their desired level of subjective effects. Outcome measures included subjective effects, autonomic effects, adverse effects, plasma hormone concentrations, and pharmacokinetics up to 3 h after starting the infusion. DMT infusions exhibited dose-proportional pharmacokinetics and rapidly induced dose-dependent subjective effects that reached a plateau after 30 min. A ceiling effect was observed for “good drug effect” at 1.8 mg/min. The 2.4 mg/min dose of DMT induced greater anxious ego dissolution than the 1.8 mg/min dose and induced significant anxiety compared with placebo. We observed moderate acute tolerance to acute effects of DMT. In the self-guided titration session, the participants opted for moderate to strong psychedelic effects, comparable in intensity to the 1.8 mg/min DMT dose rate in the randomized dosing sessions. These results may assist with dose finding for future DMT research and demonstrate that acute subjective effects of DMT can be rapidly adjusted through dose titration.

## Introduction

*N,N*-dimethyltryptamine (DMT) is a naturally occurring and short-acting psychedelic compound that is known for its potent psychoactive effects, which are primarily mediated by the activation of serotonin 5-hydroxytryptamine-2A (5-HT_2A_) receptors [1, 2]. When DMT is administered orally and alone, it is rapidly metabolized by monoamine oxidase (MAO) enzymes in the gastrointestinal tract, rendering it inactive by preventing its systemic absorption [3]. Therefore, when DMT is administered orally, it is combined with an MAO inhibitor, such as in Ayahuasca or in a synthetic combination [4]. Alternatively, DMT can be administered parenterally, thereby avoiding hepatic first-pass metabolism. Modern clinical trials that examined DMT for the treatment of various psychiatric and neurodegenerative disorders mainly used the intravenous or inhalative route [5–11]. Historically, the first trials with intravenous DMT used bolus injections [12, 13]. Recently, we tested a regimen that combined initial intravenous DMT bolus doses of 15 and 25 mg, administered within 45 s, followed by continuous infusions that were administered at dose rates of 0.6 and 1 mg/min over 90 min [5]. Another study administered bolus doses of 6–18 mg DMT fumarate over 30 s, followed by 30 min infusions of 0.6–1.9 mg/min of DMT fumarate [6]. The bolus doses produced rapidly increasing subjective effects that were often overwhelming and short-lived [5, 12, 13]. Conversely, continuous infusions at dose rates of 0.6 and 1.0 mg/min DMT without a bolus induced mild to moderate psychedelic effects that gradually reached a plateau within 20–30 min [5]. Infusions of DMT without a bolus or with a slowly administered loading dose resulted in less anxiety compared with a bolus injection and was better tolerated [5, 7, 9]. Thus, we hypothesized that continuous infusions alone may be most suitable for therapeutic use and to achieve stable, intense, but well-tolerated acute drug effects of DMT. However, acute effects and pharmacokinetics of continuous DMT infusions at different doses and over longer infusion durations are currently unknown. Therefore, the present study investigated dose-dependent acute subjective, autonomic, endocrine, and adverse effects and pharmacokinetics of intravenous DMT using random-order administration of a range of different doses. At the end of the study, we also tested a self-guided titration scheme that allowed participants to adjust the DMT dose rate at prespecified time points to achieve their desired level of subjective effects.

## Methods and materials

### Study design

The study used a double-blind, placebo-controlled, cross-over design with five randomized test sessions to investigate responses to (i) placebo, (ii) 0.6 mg/min DMT, (iii) 1.2 mg/min DMT, (iv) 1.8 mg/min DMT, and (v) 2.4 mg/min DMT. The dose selection was based on our previous study that used 0.6 and 1.0 mg/min DMT [5]. Self-guided titration was also tested in the sixth test session that was not randomized. The washout periods between sessions were at least 7 days, a duration expected to result in no carry-over effects [5]. The study was conducted in accordance with the Declaration of Helsinki and International Conference on Harmonization Guidelines in Good Clinical Practice and approved by the Ethics Committee of Northwest Switzerland (EKNZ) and Swiss Federal Office for Public Health. The study was registered at ClinicalTrials.gov (NCT05384678).

### Participants

Twenty-two healthy participants (11 men and 11 women; mean age ± SD: 30 ± 5 years; range: 25–45 years) were recruited by word of mouth or from a pool of volunteers who had contacted our research group because they were interested in participating in a clinical trial that investigated psychedelics. All participants provided written informed consent and were paid for their participation. Exclusion criteria were age <25 years or >65 years, pregnancy (urine pregnancy test at screening and before each test session), personal or family (first-degree relative) history of major psychiatric disorders (assessed by a semi-structured clinical interview based on the *Diagnostic and Statistical Manual of Mental Disorders*, 5th edition [14], by a trained physician), the use of medications that may interfere with the study medications (e.g., antidepressants, antipsychotics, or sedatives), chronic or acute physical illness (e.g., abnormal physical exam, electrocardiogram, or hematological and chemical blood analyses), tobacco smoking (>10 cigarettes/day), lifetime prevalence of psychedelic drug use >20 times, illicit drug use within the last 2 months (except for Δ^9^-tetrahydrocannabinol), and illicit drug use during the study period (screened for by urine drug tests). The participants were asked to consume no more than 20 standard alcoholic drinks/week and have no more than one drink on the day before the test sessions. Twenty participants had previously used a psychedelic, including lysergic acid diethylamide (LSD; 10 participants, 1–10 times), psilocybin (12 participants, 1–5 times), DMT (including ayahuasca; seven participants, 1–7 times), mescaline (2 participants, once), and 4-bromo-2,5-dimethoxyphenylethylamine (4 participants, 1–5 times). Twenty-one participants had previously used 3,4-methylenedioxymethamphetamine (1–50 times). Eighteen participants had previously used a stimulant, including methylphenidate (3 participants, 1–30 times), amphetamine (9 participants, 1–9 times), and cocaine (12 participants, 1–5 times). Thirteen participants had previously used ketamine (1–5 times).

### Study drugs

DMT hemifumarate (99.9% high-performance liquid chromatography purity, ReseaChem GmbH, Burgdorf, Switzerland) was formulated according to Good Manufacturing Practice and prepared in sterile vials that contained 72 mg/ml of DMT in 1 ml of purified water. The exact analytically confirmed DMT hemifumarate content of the vials (mean ± SD) was 70.34 ± 0.52 mg (*n* = 10 samples). The stability of DMT in the vials was confirmed for the study duration. Placebo consisted of identical vials that were filled with sterile water only. For the randomized sessions (study days 1–5), the content of four vials that contained either 72 mg/ml DMT or placebo was aspirated into the infusion pump syringe and diluted with saline solution (0.9% NaCl) to a volume of 50 ml. For the nonrandomized self-guided titration session, the content of three 72 mg/ml DMT vials was aspirated into the syringe and diluted with saline solution (0.9% NaCl) to a volume of 50 ml. Each participant received a continuous infusion of 50 ml over 120 min, starting at t = 0 min. In the sixth session, participants started at a dose rate of 1.2 mg/min. After 40 and 80 min, the participants were asked whether they wanted to adjust the dose rate slightly (±0.2), moderately (±0.4), or strongly (±0.6 mg/min). Thus, the maximum attainable dose rate was 2.4 mg/min, which corresponded to the highest dose that was used during the blinded study part. The participants were encouraged to self-titrate to a level of subjective effects they found the most pleasurable. At the end of each randomized session and at the end of the study, the participants were asked to retrospectively guess their treatment assignment to evaluate blinding.

### Study procedures

The study included a screening visit, five 5-h randomized test sessions, a subsequent sixth 5-h self-guided titration session, and an end-of-study visit. The sessions were conducted in a calm hospital room. Only one research participant and two investigators were present during each session. The test sessions began at ~1:00 PM, with starting times that remained consistent within each participant across all six sessions. A urine sample was taken to verify abstinence from drugs of abuse, and a urine pregnancy test was performed in women prior to each session. The participants then underwent baseline measurements. DMT or placebo was administered at 2:00 PM in sessions 1–5. Open-label DMT was administered at 2:00 PM in the sixth session. The outcome measures were repeatedly assessed for 180 and 160 min in sessions 1–5 and session 6, respectively. The participants were sent home ~15 min after the last measurement.

### Subjective drug effects

Subjective effects were assessed repeatedly using subjective effect scales 1 h before and 0, 2.5, 5, 7.5, 10, 15, 20, 30, 40, 50, 60, 70, 80, 90, 100, 110, 120, 122.5, 125, 130, 135, 140, 150,160, 170, and 180 min after starting the infusion in sessions 1–5. In session 6, subjective effects were assessed 1 h before and 0, 2.5, 5, 10, 15, 20, 30, 40, 42.5, 45, 50, 60, 70, 80, 82.5, 85, 90, 100, 110, 120, 122.5, 125, 130, 135, 140, 150, and 160 min after starting the drug administration. Subjective effect scales included verbal ratings of “any drug effect,” “good drug effect,” “bad drug effect,” and “anxiety” (Likert scale; 0–10 for no to maximal effect). The 5 Dimensions of Altered States of Consciousness (5D-ASC) scale [15, 16] was administered at the end of each test session. Mystical experiences were assessed at the end of each study session using the States of Consciousness Questionnaire (SOCQ) [17–19] that includes the 30-item Mystical Effects Questionnaire (MEQ30) [20], the 40-item Mystical Effects Questionnaire (MEQ40), and the 48-item Psychedelic Experience Scale 48 (PES48) [19]. Subjective effect measurements are described in more detail in the Supplementary Methods online.

### Autonomic and adverse effects

Blood pressure and heart rate were measured at baseline and 2.5, 7.5, 15, 20, 30, 40, 60, 80, 100, 120, 125, 130, 135, 140, 150,160, 170, and 180 min after the start of the infusion in sessions 1–5. In session 6, blood pressure and heart rate were measured at baseline and 10, 30, 50, 70, 90, 110, 130, and 150 min after the start of the infusion. Adverse effects were assessed 1 h before and 180 and 160 min after drug administration in sessions 1–5 and the sixth session, respectively, using the List of Complaints [21].

### Endocrine effects and brain-derived neurotrophic factor levels

Plasma concentrations of oxytocin, cortisol, and prolactin and serum concentrations of brain-derived neurotrophic factor (BDNF) were determined as previously described [22, 23] in sessions 1–5. Oxytocin, cortisol, and prolactin were measured at baseline and 110 and 180 min after the start of the infusion. BDNF was measured at baseline and 110 min after the start of the infusion.

### Plasma DMT concentrations

Blood was collected into lithium heparin tubes at baseline and 2.5, 5, 7.5, 10, 15, 20, 30, 40, 50, 60, 70, 80, 90, 100, 110, 120, 122.5, 125, 130, 135, 140, 150, 160, 160, 170, and 180 min after the start of the infusion in sessions 1–5. The blood samples were immediately centrifuged, and plasma was subsequently stored at −20 °C and −80 °C until analysis. Plasma concentrations of DMT and metabolites were determined by high-performance liquid chromatography-tandem mass spectrometry using a validated method as previously described [24].

### Pharmacokinetic analyses

Pharmacokinetic parameters were estimated using non-compartmental methods as described previously [5, 25]. Two participants who stopped the infusion prematurely were excluded from the pharmacokinetic analysis. Analyses were conducted using Phoenix WinNonlin 8.4 (Certara, Princeton, NJ, USA).

### Data analysis

Peak (E_max_) or peak change from baseline (ΔE_max_) values were determined for repeated measures. The values were then analyzed using analysis of variance (ANOVA), with dose as the within-subjects factor, followed by the Tukey *post hoc* test, using R 4.2.1 software. The criterion for significance was *p* < 0.05.

## Results

### Subjective drug effects

**Fig. 1:**
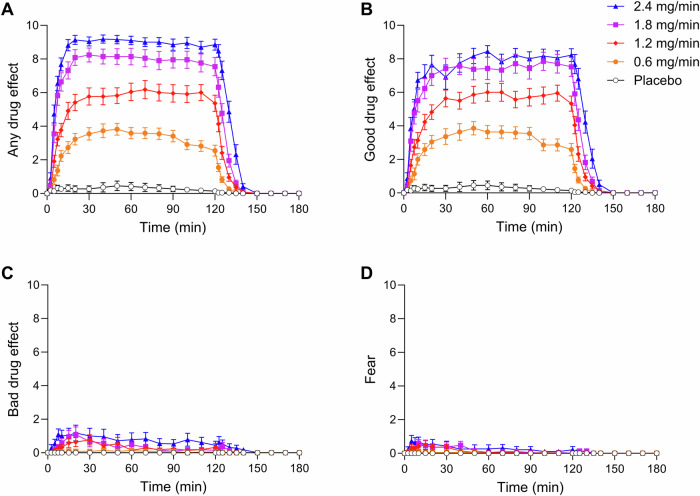
Acute subjective effects of *N,N*-dimethyltryptamine (DMT) over time. The different intravenous dose rates were the following: 0.6, 1.2, 1.8, and 2.4 mg/min. All infusion rates induced “any drug effect” (**A**) and “good drug effect” (**B**) that reached peak effects after 30 min. DMT dose-dependently elicited “good drug effect” with a ceiling at a dose rate of 1.8 mg/min. The 2.4 mg/min dose rate did not significantly increase “good drug effect” (**B**) compared with the 1.8 mg/min dose rate, but it significantly increased “bad drug effect” (**C**) and “fear” (**D**) compared with 0.6 mg/min and placebo. After stopping the infusion, all drug effects rapidly and completely subsided within 20 min. Infusions were started at *t* = 0 min and stopped at *t* = 120 min. The data are expressed as the mean ± SEM in 22 healthy participants. The corresponding maximal responses and statistics are shown in Supplementary Table S1.

Subjective effects over time on the subjective effect scales in sessions 1–5 are shown in Fig. 1. Statistics are summarized in Supplementary Table S1. DMT dose-dependently elicited subjective responses that were significantly different from placebo. A ceiling effect was observed at the 1.8 mg/min dose rate for “good drug effect” (Fig. 1B). Conversely, the 2.4 mg/min dose rate further increased “bad drug effect” and “fear” compared with the 0.6 mg/min dose and placebo (Fig. 1C, D). The infusion was stopped three times in two participants prematurely after 20–40 min because of overwhelming psychedelic effects and intense fear (twice at 2.4 mg/min and once at 1.8 mg/min). Both participants had prior experience with psychedelics. Infusions of 1.2, 1.8, and 2.4 mg/min induced gradually increasing psychedelic effects that reached plateaus after 30 min and remained stable at their respective levels until the infusion ended at 120 min. In contrast, subjective effects of the lowest dose rate (0.6 mg/min) slightly and gradually decreased between 50 and 120 min. After stopping the infusion, all drug effects rapidly and completely subsided within 20 min.

**Fig. 2:**
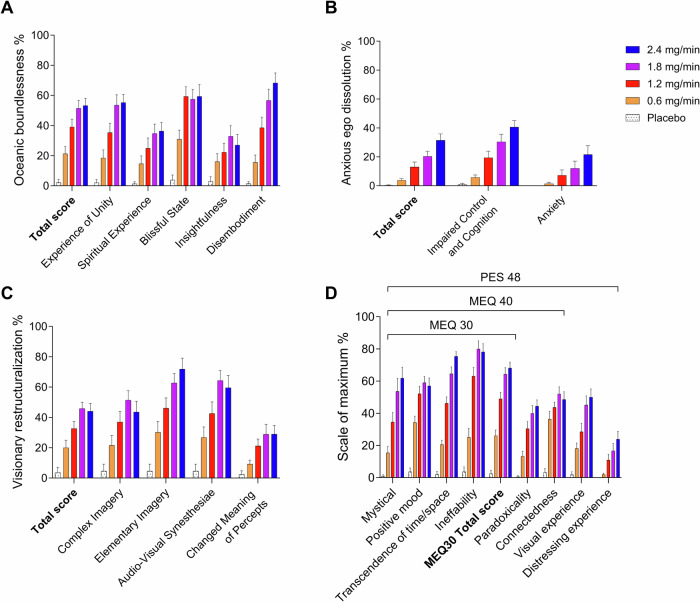
Acute mind-altering and mystical effects of N,N-dimethyltryptamine (DMT). Acute alterations of mind on the 5 Dimensions of Altered States of Consciousness (5D-ASC) scale (**A**–**C**) and acute mystical-type experiences on the Mystical Experience Questionnaire 30 (MEQ30), Mystical Experience Questionnaire 40 (MEQ40), and Psychedelic Experience Scale 48 (PES48) (**D**) that were induced by *N,N*-dimethyltryptamine (DMT). DMT produced dose-dependent alterations of consciousness and mystical-type effects compared with placebo. A ceiling effect was observed for oceanic boundlessness and visionary restructuralization on the 5D-ASC and for mystical-type effects on the MEQ30 at the 1.8 mg/min dose rate. In contrast, ratings of anxious ego dissolution further increased at 2.4 mg/min compared with the 1.8 mg/min dose rate. Additionally, only the 2.4 mg/min dose rate induced significant anxiety compared with placebo. The data are expressed as the mean ± SEM in 22 healthy participants. Statistics are shown in Supplementary Table S2.

Alterations of mind and mystical-type effects during sessions 1–5 are shown in Fig. 2. Statistics are summarized in Supplementary Table S2. All DMT infusions induced pronounced psychedelic effects compared with placebo, including full mystical experiences (≥60% on all four subscales of the MEQ30) in two, two, and five participants in the 1.2, 1.8, and 2.4 mg/min dose rate conditions, respectively (Fig. 2D). A ceiling effect was reached at the 1.8 mg/min dose rate of DMT on most scales of the 5D-ASC and MEQ30, with no significant differences between the 1.8 and 2.4 mg/min doses (Fig. 2). However, the 2.4 mg/min dose produced significantly greater anxious ego dissolution on the 5D-ASC (Fig. 2B) compared with the 1.8 mg/min dose (*p* < 0.05). Moreover, anxiety significantly increased at 2.4 mg/min compared with placebo and 0.6 and 1.2 mg/min DMT (Fig. 2B). Thus, mostly negative subjective effects further increased at the highest dose rate of 2.4 mg/min (Supplementary Tables S1 and S2).

### Autonomic and adverse effects

Autonomic effects over time and related peak effects are shown in Supplementary Fig. S5 and Supplementary Table S1, respectively. DMT dose-dependently increased systolic and diastolic blood pressure compared with placebo. Heart rates were significantly elevated at dose rates of 1.2 mg/min or above (Supplementary Fig. S5C). Only the 2.4 mg/min dose rate increased adverse effects compared with placebo (Supplementary Table S1). Frequencies of specific adverse effects are presented in Supplementary Table S3. Frequent complaints included headache, tiredness, impaired concentration, feeling of weakness, hot flashes, and palpitations. One serious adverse event, possibly related to DMT, involved a participant who discontinued the study due to the exacerbation of preexisting tinnitus. Another participant discontinued the study after two sessions (1.8 and 2.4 mg/min) due to anxiety about facing potentially higher doses in later sessions. Both participants had prior experience with psychedelics.

### Endocrine levels

DMT dose-dependently increased serum prolactin and cortisol concentrations (Supplementary Fig. S1 and Supplementary Table S1). Dose rates of 1.2 mg/min and above significantly increased serum prolactin, and 1.8 mg/min and above increased cortisol compared with placebo. None of the DMT dose rates altered serum BDNF or plasma oxytocin levels (Supplementary Fig. S2 and Supplementary Table S1). Moreover, serum BDNF levels did not increase between sessions 1 and 5 (Supplementary Fig. S3).

### Pharmacokinetics and pharmacokinetic-pharmacodynamic relationship

**Fig. 3:**
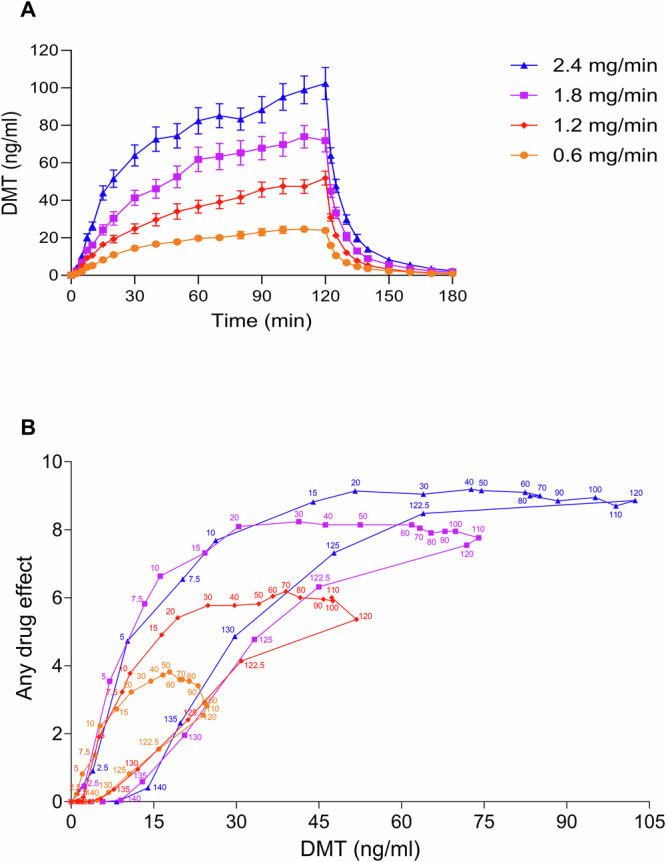
Pharmacokinetics and pharmacokinetic-pharmacodynamic relationship of *N,N*-dimethyltryptamine (DMT). Plasma DMT concentration-time curves for 0.6, 1.2, 1.8, and 2.4 mg/min doses of DMT (**A**) are expressed as the mean ± SEM in 22 healthy participants. Plasma DMT concentrations increased proportionally with increasing dose rates. Plasma concentrations increased rapidly within the first 30 min across all dose rates. From 30 to 120 min, the plasma concentration continued to increase slowly and gradually; thus, steady-state levels were not attained at any of the administered dose rates. After stopping the infusion at 120 min, DMT levels very rapidly decreased within 20 min in all conditions. The corresponding pharmacokinetic parameters are shown in Table 1. Individual concentrations are shown in Supplementary Fig. S6. **B** Plasma concentration-response relationships for 0.6, 1.2, 1.8, and 2.4 mg/min dose rates of DMT in 22 healthy participants. Plasma concentration values are expressed as the mean, and responses are expressed as the mean on the subjective effect scale of “any drug effect.” The time of sampling (in minutes) is indicated next to each data point. All doses of DMT showed moderate clockwise hysteresis, indicating acute pharmacological tolerance.

Table 1 shows pharmacokinetic parameters of DMT based on noncompartmental analyses. Concentration-time and concentration-effect curves of DMT are shown in Fig. 3. Individual concentration-time curves are shown in Supplementary Fig. S6. Concentration-time curves of DMT on a semilogarithmic plot are shown in Supplementary Fig. S7. Concentration-time curves of the main metabolites indole-3-acetic acid and DMT-*N*-oxide are shown in Supplementary Fig. S4. Plasma DMT concentrations increased proportionally with increasing doses (Table 1 and Fig. 3). The mean maximum plasma concentrations (C_max_) for the 0.6, 1.2, 1.8, and 2.4 mg/min dose rates were 26, 51, 78, and 105 ng/ml, respectively, and were reached (T_max_) after 86, 102, 96, and 96 min, respectively. Two distinct plasma half-lives were observed after stopping the infusion at 120 min: an early half-life (t_1/2α_) with a mean value of 6.3–7.1 min and a late half-life (t_1/2β_) with a mean value of 18–19 min (Table 1). The switch from early to late elimination occurred 20 min after stopping the infusion and at mean DMT concentrations of 2.0, 3.6, 6.5, and 9.0 ng/ml for the 0.6, 1.2, 1.8, and 2.4 mg/min dose rates, respectively.

**Table 1:** Pharmacokinetic parameters for DMT based on non–compartmental analyses [geometric mean (95% CI), range].

	0.6 mg/min	1.2 mg/min	1.8 mg/min	2.4 mg/min
C_max_ (ng/ml)	26 (22–31)	51 (42–61)	78 (63–96)	105 (85–128)
	13–46	23–88	20–149	24–174
T_max_ (min)	86 (74–99)	102 (90–116)	96 (85–108)	96 (81–114)
	40–120	50–120	50–120	40–120
t_1/2α_ (min)	6.3 (5.6–7.2)	6.4 (5.7–7.2)	6.8 (6.1–7.6)	7.1 (6.1–8.3)
	3.9–9.9	4.3–7.2	4.0–9.9	4.3–16
t_1/2β_ (min)	19 (16–23)	18 (17–20)	18 (16–19)	18 (17–20)
	9.8–44	14–26	13–29	14–24
CL (L/min)	34 (28–41)	36 (29–44)	34 (27–43)	32 (26–39)
	20–78	18–106	17–158	19–132
V_z_ (L)	912 (704–1183)	941 (743–1192)	862 (663–1121)	832 (654–1058)
	342–2047	373–2682	382–3346	466–3774
AUC_∞_ (ng*min/ml)	2153 (1767–2623)	4055 (3322–4950)	6342 (5043–7977)	9098 (7392–11199)
	934–3592	1371–7905	1375–12409	2195–15249
Start time of t_1/2β_ (min)	141 (139–144)	142 (140–145)	142 (139–144)	143 (141–145)
	130–152	135–151	135–150	135–150
DMT concentration at the start of t_1/2β_ (ng/ml)	2.0 (1.6–2.5)	3.6 (2.6–4.8)	6.5 (4.8–8.6)	9.0 (6.8–11.9)
	0.82–6.9	1.05–10.9	2.1–18.7	2.9–19.9

*AUC* area under the plasma concentration-time curve, *AUC*_*∞*_ AUC from time zero to infinity, *CL* apparent total clearance, *C*_*max*_ maximum observed plasma concentration, *t*_*1/2α*_ early elimination plasma half-life, *t*_*1/2β*_ late elimination plasma half-life, *T*_*max*_ time to reach C_max_, *95%CI* 95% confidence interval, *V*_*z*_ apparent volume of distribution; *n* = 20.

The DMT concentration-response relationship over time is shown in Fig. 3. We observed moderate acute tolerance to subjective effects of DMT, indicated by clockwise hysteresis (Fig. 3). Specifically, subjective effects showed no increase from 30 to 120 min, despite increasing plasma concentrations. At a dose rate of 0.6 mg/min, subjective effects gradually decreased from 50 to 120 min.

### Blinding

Data on the participants’ retrospective identification of the DMT dose conditions are shown in Supplementary Table S4. Most conditions were correctly identified by the participants at the end of the study. However, immediately after a study session, placebo, and the 0.6, 1.2, 1.8, and 2.4 mg/min dose rates were misclassified by 3 (14%), 5 (23%), 8 (36%), 11 (50%), and 7 (32%) participants, respectively. In most of these cases, the conditions were confounded with the next higher or lower dose rate.

### Self-guided titration (study day 6)

**Fig. 4:**
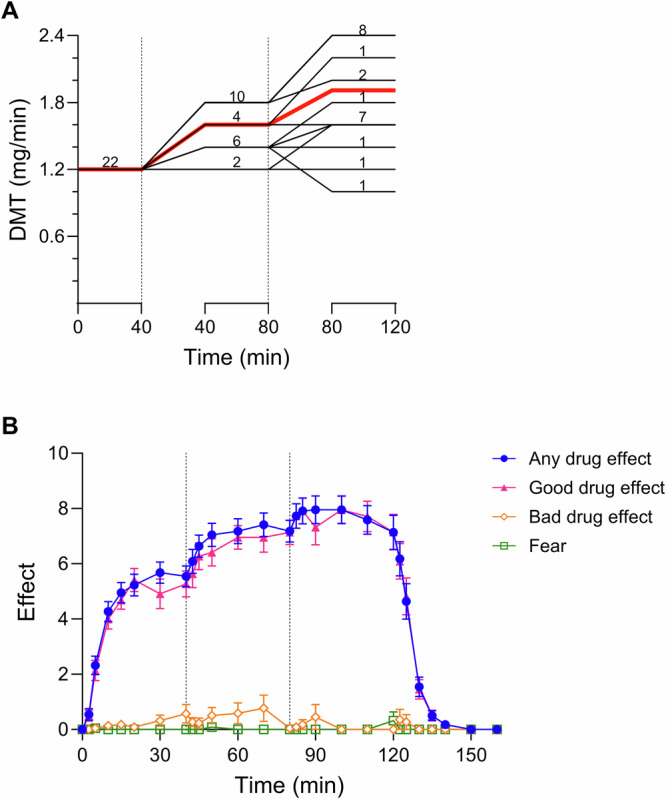
Self-guided titration of *N,N*-dimethyltryptamine (DMT). **A** Dose rates of DMT over time. The black lines represent the individual dose rates, and the mean is indicated in red. The numbers on the black lines represent the number of participants at each dose rate and time. **B** Acute subjective effects over time. Participants increased the dose to a mean dose rate of 1.9 mg/min, achieving similar levels of “any drug effect” compared with the 1.8 mg/min condition in the randomized session. Ratings of “bad drug effect” were low, and “fear” was practically absent. Subjective effects quickly responded within 5–10 min following dose-adjustments. DMT infusions started at *t* = 0 min with a dose rate of 1.2 mg/min. Participants could adjust the dose rate at t = 40 min and t = 80 min in steps of ±0.2, ±0.4, or ±0.6 mg/min. Infusions were stopped at *t* = 120 min. The data are expressed as the mean ± SEM in 22 healthy participants.

Individual doses and corresponding subjective effects over time of the sixth study day are shown in Fig. 4. In session 6, the participants had the possibility to self-titrate to the level of subjective effects they found most desirable. The participants started at a moderate dose rate of 1.2 mg/min and could change the rate after 40 and 80 min. The participants opted for strong psychedelic effects, reflected by a peak “any drug effect” of 8.2 ± 0.5 (mean ± SD) on the subjective effect scale. Ratings of “bad drug effect” and “fear” were very low (Fig. 4). The subjective effects adapted rapidly within 5–10 min after a dose adjustment. The administered DMT dose rates ranged from 1.0 to 2.4 mg/min. Approximately one-third of the participants increased the dose to the maximum DMT dose rate of 2.4 mg/min, and another third increased the dose rate to 1.6 mg/min. One participant did not change the starting dose rate of 1.2 mg/min, and one reduced the rate to 1 mg/min (Fig. 4).

## Discussion

The present study characterized acute dose-dependent effects and pharmacokinetics of intravenous DMT over an infusion duration of 120 min in healthy participants. We found that DMT dose-dependently produced acute subjective effects that reached a ceiling effect at a dose rate of 1.8 mg/min with regard to its positive subjective drug effects. However, the 2.4 mg/min dose rate produced significantly greater anxious ego dissolution compared with the 1.8 mg/min dose rate and more “fear” and “bad drug effect” compared with the 0.6 mg/min dose rate and placebo. Additionally, two participants decided to stop the infusion at the 2.4 mg/min dose rate because of overwhelming psychedelic effects and intense fear. One of the two participants also stopped the infusion at the 1.8 mg/min dose rate. The infusions were stopped relatively early after 20–40 min, indicating that the initial rapid increase in subjective effects may be particularly challenging. Similarly, we previously found that intravenous bolus doses are less well tolerated than continuous infusions, in which the onset of effects is even more rapid [5].

Previous research on intravenous DMT examined the combination of an initial bolus dose with a continuous infusion [5, 6]. However such high bolus doses were less well tolerated [5, 12, 13] than continuous infusions [5, 7] likely because of greater negative drug effects, including fear and physical discomfort, that are associated with the rapid and immediate onset of acute effects of bolus doses [5]. Inhalation of high doses of DMT, which exhibits kinetics similar to that of intravenous bolus doses, may be associated with similar tolerability issues [11]. Conversely, the presently tested continuous infusion schedules without an initial bolus induced gradually increasing psychedelic effects that reached a plateau relatively quickly within 20–30 min and were very well tolerated, which is consistent with a previous study that used dose rates of 0.6 and 1.0 mg/min [5]. Thus, continuous infusions alone may be sufficient to gradually and safely achieve strong psychedelic effects within 20–40 min without requiring an initial bolus dose. This means that strong but also well-tolerated psychedelic states can be induced within a relatively short session. Thus, this intervention is a possible cost-effective therapeutic option in contrast to the administration of other psychedelics, such as psilocybin and LSD, which have acute effects that last up to 6 and 12 h, respectively [26, 27]. Moreover, the present study found that intravenous infusions of DMT can be terminated whenever desired, resulting in a fast return to normal mental states. This represents an advantage of intravenous DMT over psilocybin and LSD, which have much longer plasma elimination half-lives [26, 27] and thus longer-lasting acute effects and recovery times, even if they are administered intravenously. While the close analog 5-Methoxy-DMT shares the advantage of a short elimination half-life, it exhibits notable differences in receptor interactions and subjective effects compared with other classical psychedelics such as psilocybin, LSD, and DMT [28, 29].

The present study documented the presence of acute pharmacological tolerance to the continuous administration of DMT. All dose rates induced rapidly and dose-dependently increasing subjective effects within 20–30 min when a plateau was reached until the infusion was ended at 120 min. At the 0.6 mg/min dose rate, subjective effects even slightly decreased from 50 to 120 min. Conversely, plasma DMT concentrations increased rapidly within 30 min but then continued to further increase from 30 to 120 min by ~40%. Thus, steady-state concentrations of DMT were not attained at any of the administered dose rates over 120 min, confirming and expanding similar previous findings with continuous infusions over 90 min [5]. The observation that subjective effects remained stable or even slightly decreased while plasma DMT concentrations were still rising was further indicated by clockwise hysteresis in the concentration-effect curve over time and is consistent with previous findings [5, 6]. In contrast, no tolerance to subjective effects of DMT were found with repeated bolus administrations of DMT within the same day [30]. Our findings and others [5, 6] do not support the view that DMT exerts no tolerance, in contrast to other psychedelics [30]. In fact, little to no acute pharmacological tolerance is observed with LSD, psilocybin, or mescaline. The observation of tolerance or no tolerance may thus depend on the route of administration (intravenous bolus *vs*. intravenous continuous infusion or oral administration), the nature of the response (rapidly spiking and short lasting *vs*. steady increase and decline), and methodological aspects (measurement of peak response *vs*. effect-concentration time curves).

The present study also validly determined pharmacokinetic parameters of DMT across a large dose range using concentration measurements after stopping the infusion. DMT was very rapidly cleared from plasma, with an initial half-life (t_1/2α_) of 6.3–7.1 min over 20 min until plasma DMT concentrations of 2.0–9.0 ng/ml were reached for the 0.6–2.4 mg/min dose rates. The plasma concentration then further decreased more slowly, with a longer late plasma half-life (t_1/2β_) of 18–19 min. Importantly, the subjective effects rapidly declined in parallel with the initial short plasma half-life within 20 min, indicating that initial elimination is more clinically relevant. The observed initial half-life was similar but slightly longer than previously described (t_1/2α_ = −5.0–5.8 min) [5]. In the present study, we included an additional plasma concentration measurement that was taken 2.5 min after the end of the infusion. This additional measurement likely enabled a more precise estimation of t_1/2α_. Another study reported a longer mean plasma half-life of DMT of 9–12 min in 24 subjects [9]. However, no distinction was made between an early short plasma half-life and a longer late plasma half-life in that analysis. Additionally, the infusion duration was only 10 min and included an initial bolus dose. The rapid early elimination reflects the MAO activity and very rapid metabolism of DMT. The switch to a longer plasma half-life after 20 min at different plasma DMT concentrations suggests the presence of distribution kinetics with varying degrees of DMT redistribution from tissue and the intracellular space back into the circulation, depending on the amount infused. As such, higher dose rates may result in higher tissue concentrations of DMT and thus more extensive redistribution into the circulation. These considerations are primarily important for the conceptualization of possible compartmental models for the pharmacokinetics of DMT, which we did not establish for the present publication. Moreover, the proposed distributional processes might explain the steadily rising plasma concentrations from 30 to 120 min that seem most pronounced at higher dose rates. Furthermore, evidence of distribution kinetics has been previously described [5, 31] and may further account for the variability in estimates of the elimination half-life of DMT [5, 9, 12, 13].

In the present study, DMT dose-dependently increased serum cortisol and prolactin levels, which are markers of serotonergic activity [32, 33], similar as previously reported for intravenous bolus doses [13] and other psychedelics [27, 33]. We observed no increase in serum BDNF levels, consistent with our previous findings with DMT [5] and LSD and psilocybin [27]. Moreover, oxytocin levels were not altered. In a previous study, oxytocin levels were slightly elevated at a dose rate of 1 mg/min DMT compared with placebo [5]. However, the lack of a significant oxytocin elevation across a wide dose range in the present study suggests that DMT does not induce clinically relevant oxytocin release, in contrast to LSD [26, 27] and mescaline [26].

DMT administration resulted in moderate, dose-dependent, and transient increases in arterial blood pressure and heart rate. The observed cardiostimulant effects of DMT were slightly more pronounced compared indirectly with other psychedelics, such as LSD and psilocybin [23, 27].

As expected for a double-blind study using different psychoactive doses [23], blinding between different DMT doses was relatively well maintained. When assessed after the study sessions, the different conditions were misclassified by the participants in approximately one-third of the cases. In most cases of misclassification, the conditions were confounded with the next higher or lower dose rate. The present study may provide support for the selection of low and minimally active DMT doses for an “active placebo” arm in later therapeutic trials. For example, the 0.6 mg/min dose of DMT could be used as an additional control group to enhance blinding in a therapeutic trial using a higher potentially therapeutically active dose (1.8 mg/min) and an inactive placebo control condition.

The present study was the first to evaluate the self-titration of a psychedelic in a formalized controlled study setting. In the last open-label DMT study session, the participants had the possibility to self-titrate to the level of subjective effects they found most desirable. On average, the participants opted for strong psychedelic effects, comparable in average intensity to the 1.8 mg/min dose rate in the blinded randomized session. Importantly, negative subjective effects and particularly ratings of “fear” were very low despite strong psychedelic effects when the participants self-titrated the doses. Several aspects may explain the very good tolerability of self-guided titration. First, all participants began with a moderate dose rate of 1.2 mg/min and were permitted to increase the dose rate by a maximum of 0.6 mg/min per escalation. This approach led to a more gradual increase in subjective effects compared with the fixed 1.8 and 2.4 mg/min dose rates that were used in the randomized sessions. Second, the titration session occurred on the last study day. Therefore, the participants were experienced with DMT’s effects. Prior experience with psychedelics has been associated with lower ratings on the 5D-ASC including negative effects such as “anxious ego dissolution” and “impaired control and cognition” and may likely have improved the tolerability of the DMT administration in the present study [34, 35]. Lastly, the possibility to control the intensity of psychedelic effects in an otherwise rather uncontrollable psychological state might have provided reassurance to the participants. Notably, subjective effects adapted quickly within 5–10 min after a dose adjustment. Therefore, a more rapid dose escalation every 15–20 min may also be feasible compared with the 40-min intervals that were used in this study. Moreover, a higher starting dose rate than 1.2 mg/min could be considered for individuals with prior experience and good tolerability of DMT. To our knowledge, this is the first study to successfully test the self-guided titration of any psychedelic. However, dose titration is commonly used in clinical practice to improve the efficacy and tolerability of drug treatments and is also standard practice for recreational substance users. DMT is particularly suitable for such an application because of its very short elimination half-life, which allows for a very quick modification of subjective effects. Moreover, many psychedelic drugs, including DMT, exhibit substantial interindividual pharmacokinetic and pharmacodynamic variability [5, 6, 9, 23, 27]. Additionally, predicting the acute subjective response to psychedelics is challenging, with large parts of the variability remaining unexplained [34, 35]. Thus, the within-session self-titration of DMT dose may be a promising method to tailor dosing and subjective effects to individual needs of patients.

The present study has several notable strengths. Four different doses of DMT were administered to participants in a within-subjects design and compared with placebo under double-blind conditions in a controlled laboratory setting. Thus, we were able to accurately describe the dose-response relationship of continuous intravenous DMT across a wide dose range and over a relatively long infusion duration of 120 min. We also included equal numbers of male and female participants and used internationally established standardized and validated psychometric outcome measures. Moreover, we added a self-guided titration scheme after the blinded and random study phase, which could be used in the future to individualize treatment based on patients’ needs and therapeutic sessions.

Notwithstanding these strengths, the present study also has limitations. Self-titration occurred in participants with recent DMT experience and would need to be replicated in non-experienced individuals. The study used a highly controlled setting and included only healthy participants. Thus, subjects in different environments and patients with psychiatric disorders may respond differently to DMT.

## Conclusion

DMT infusions exhibited dose-proportional pharmacokinetics and induced dose-dependent subjective effects that reached a plateau after 30 min and rapidly declined after ending the infusion. A ceiling effect was observed for “good drug effect” at the 1.8 mg/min dose rate but not for anxious ego dissolution. The self-guided titration of DMT dosing rates during an infusion could be used in the future to tailor dosing and subjective effects to the individual needs of patients.

## Supplementary information

Supplemental Material

Table S1

Table S2

## Data Availability

The datasets presented in this article are not readily available because the data associated with this work are owned by the University Hospital Basel and were licensed by Mind Medicine. Requests to access the datasets should be directed to MEL, matthias.liechti@usb.ch.
