# Supplementary material for: Acute dose-dependent effects and self-guided titration of continuous N,N-dimethyltryptamine infusions in a double-blind placebo-controlled study in healthy participants
Source: Neuropsychopharmacology. 2024 Dec 19;50(6):1008–16. doi: 10.1038/s41386-024-02041-8 (PMC12032411; doi:10.1038/s41386-024-02041-8)
Supplement: Supplementary file 1 — Supplemental Material [file 41386_2024_2041_MOESM1_ESM.docx]

**Supplement**

**Methods**

*Subjective effect scales (SES)*

Participants were asked by the investigator to repeatedly rate their subjective effects verbally on four single-item Likert scales from 0 to 10 for: “any drug effect”, “good drug effect”, “bad drug effect”, and “fear”. Intervals were 1 h before and 0, 2.5, 5, 7.5, 10, 15, 20, 30, 40, 50, 60 70, 80, 90, 100, 110, 120, 122.5, 125, 130, 140, 150, 160, 170, and 180 minutes after drug administration. A similar method was used previously to assess DMT effect [1] and is less demanding than completing self-rated single-item visual rating scales (VAS) in written form [2] and therefore interferes only minimally with the subjective experience. The SES “any drug effect” is an overall effect measure to characterize the overall effect intensity and time course. The SES “good drug effect” is an overall measure of effects subjectively considered positive. The SES “bad drug effect” is an overall measure of any negative effects and related to “fear”. SES scales were assessed each time DMT blood concentrations were measured to allow for pharmacokinetic-pharmacodynamic modeling.

*5 Dimensions of Altered States of Consciousness (5D-ASC) scale*

The 5 Dimensions of Altered States of Consciousness (5D-ASC) scale [3,4] was used as the primary outcome measure and was administered 150 minutes after drug administration to retrospectively rate peak drug effects. The 5D-ASC scale measures altered states of consciousness and contains 94 items (visual analog scales). The instrument consists of five subscales/dimensions [3] and 11 lower-order scales [4]. The 5D-ASC dimension “Oceanic Boundlessness” (27 items) measures derealization and depersonalization associated with positive emotional states, ranging from heightened mood to euphoric exaltation. The corresponding lower-order scales include “experience of unity,” “spiritual experience,” “blissful state,” “insightfulness,” and “disembodiment.” The dimension “Anxious Ego Dissolution” (21 items) summarizes ego-disintegration and loss of self-control phenomena associated with anxiety. The corresponding lower-order scales include “impaired control of cognition” and “anxiety.” The dimension “Visionary Restructuralization” (18 items) consists of the lower-order scales “complex imagery,” “elementary imagery,” “audio-visual synesthesia,” and “changed meaning of percepts.” Two additional dimensions describe “Auditory Alterations” (15 items) and “Reduction of Vigilance” (12 items). The total 3D-ASC score is the total of the three main dimensions “Oceanic Boundlessness”, “Anxious Ego-Dissolution”, and “Visionary Restructuralization” and can be used as a measure of the overall intensity of the alteration of the mind [5]. The scale is well-validated in German [3] and many other languages and widely used to characterize the subjective effects of various psychedelic drugs. In particular, the scale has been used by most research groups to psychometrically assess LSD effects [6-11]. Furthermore, acute ratings on the 5D-ASC after administration of psilocybin have been used to predict long-term effects of psychedelic treatments in patients [12,13]. Ratings on the 5D-ASC have been shown to closely correlate with ratings on the Mystical Effects Questionnaire (MEQ, see below) [5] which is primarily used by research groups in the US [13].

*Psychedelic Experience Scale (PES48) and Mystical Effects Questionnaire (MEQ30)*

The Psychedelic Experience Questionnaire/Scale (PES48) was administered 24 h after LSD administration to retrospectively rate peak subjective effects. The PES48 [14] is a revalidation of the original 100-item States of Consciousness Questionnaire (SOCQ) [5,15] and includes the 30-item Mystical Effects Questionnaire (MEQ30) [16], as well as additional subscales. The MEQ30 consists of the subscales “mystical experience”, “positive mood”, “transcendence of time and space”, and “ineffability”. Their sum provides the MEQ30 total score, which reflects the overall intensity of the mystical experience. The MEQ30 has been used in numerous experimental trials with LSD [7,9,17-20]. Additional subscales of the PES48 assess “paradoxicality” and “connectedness,” “visual experience” and “distressing experience”. The published German version was used [5,14]. It should be noted that participants completed the full 100-item questionnaire, from which 48 items are used to derive the validated subscales [14]. Future studies could consider administering only these 48 items for analysis.

**Results**


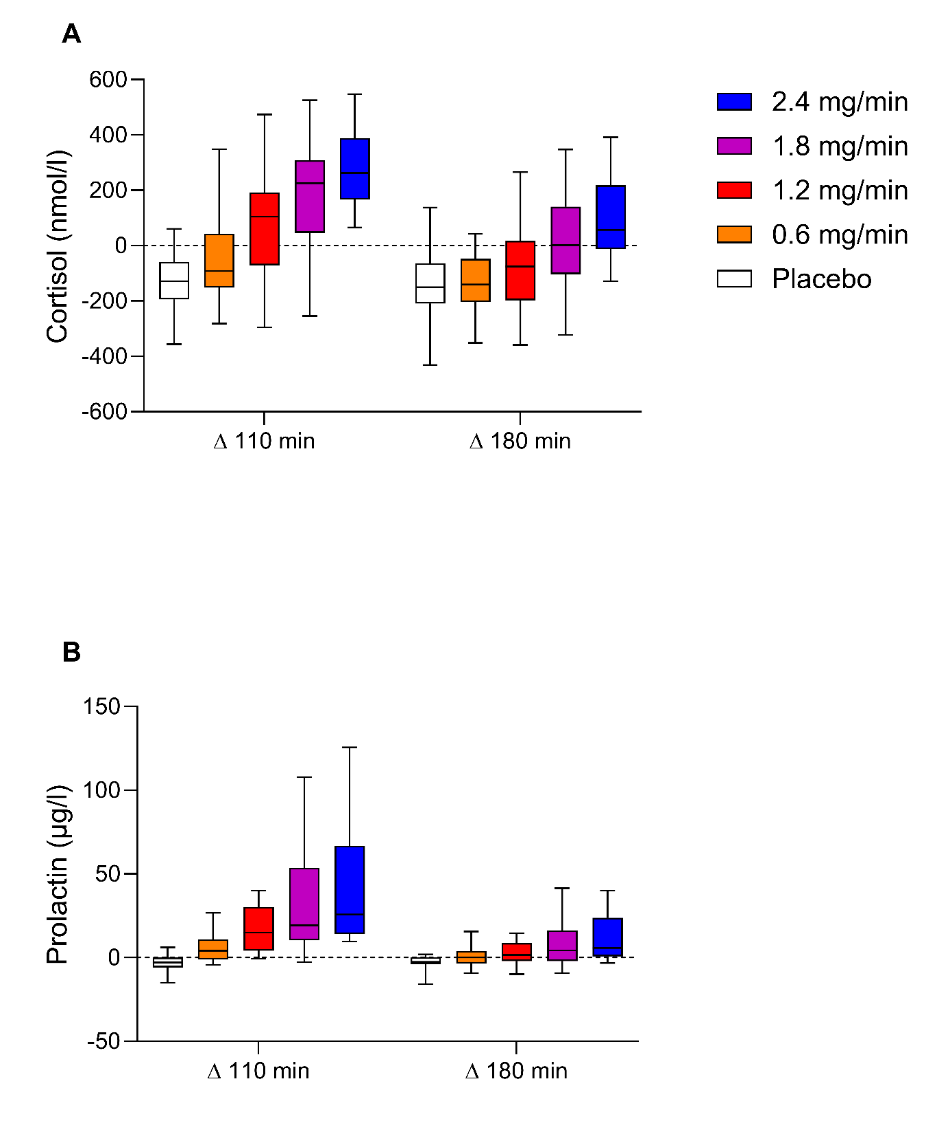


**Figure S1.** Serum concentrations of cortisol **(A)** and prolactin **(B)** at baseline (0 min) and at 110 and 180 min in 22 healthy participants. The corresponding statistics are shown in Table S1.


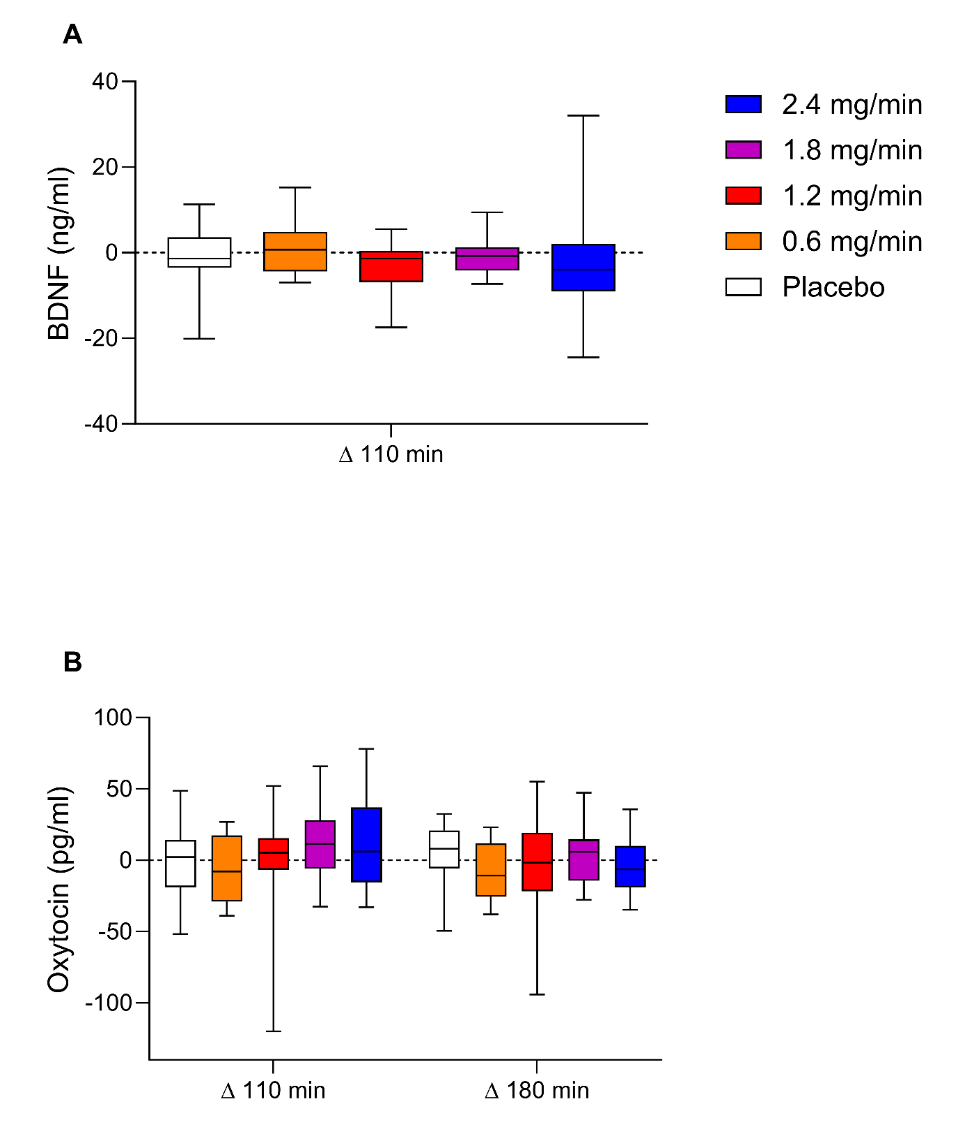


**Figure S2.** Plasma concentrations of oxytocin **(A),** showing the difference from baseline at 110 and 180 min. Serum concentrations of brain-derived neurotrophic factor (BDNF) **(B)**, showing the difference from baseline at 110 min in 22 healthy participants. The corresponding statistics are shown in Table S1.

**
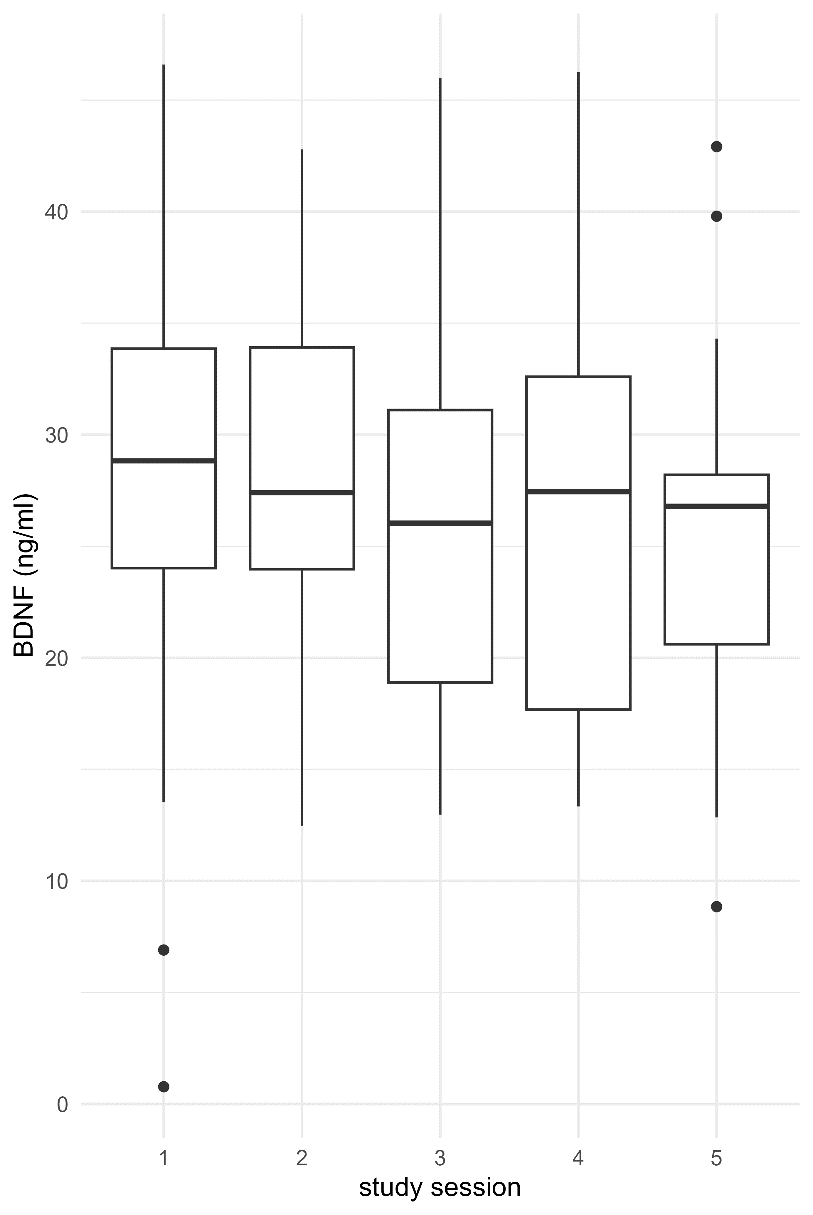
**

**Figure S3.** Serum concentrations of brain-derived neurotrophic factor (BDNF) at baseline of study sessions 1–5 in 22 healthy participants. Serum BDNF levels did not increase between sessions 1–5.


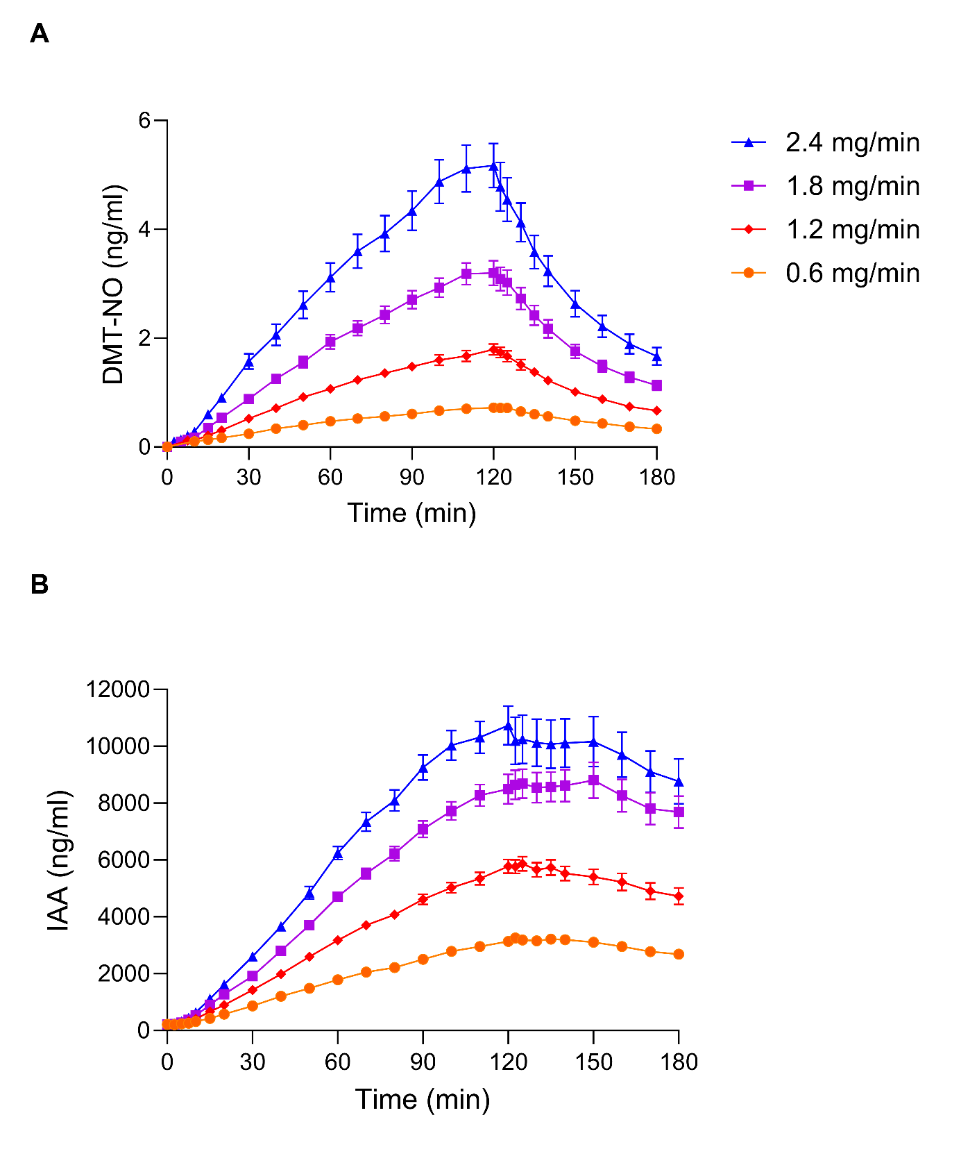


**Figure S4.** Plasma concentrations of **(A)** DMT-*N*-oxide (DMT-NO) and indole-3-acetic acid (IAA) **(B)** over time. The data are expressed as means ± SEM in 22 healthy participants.


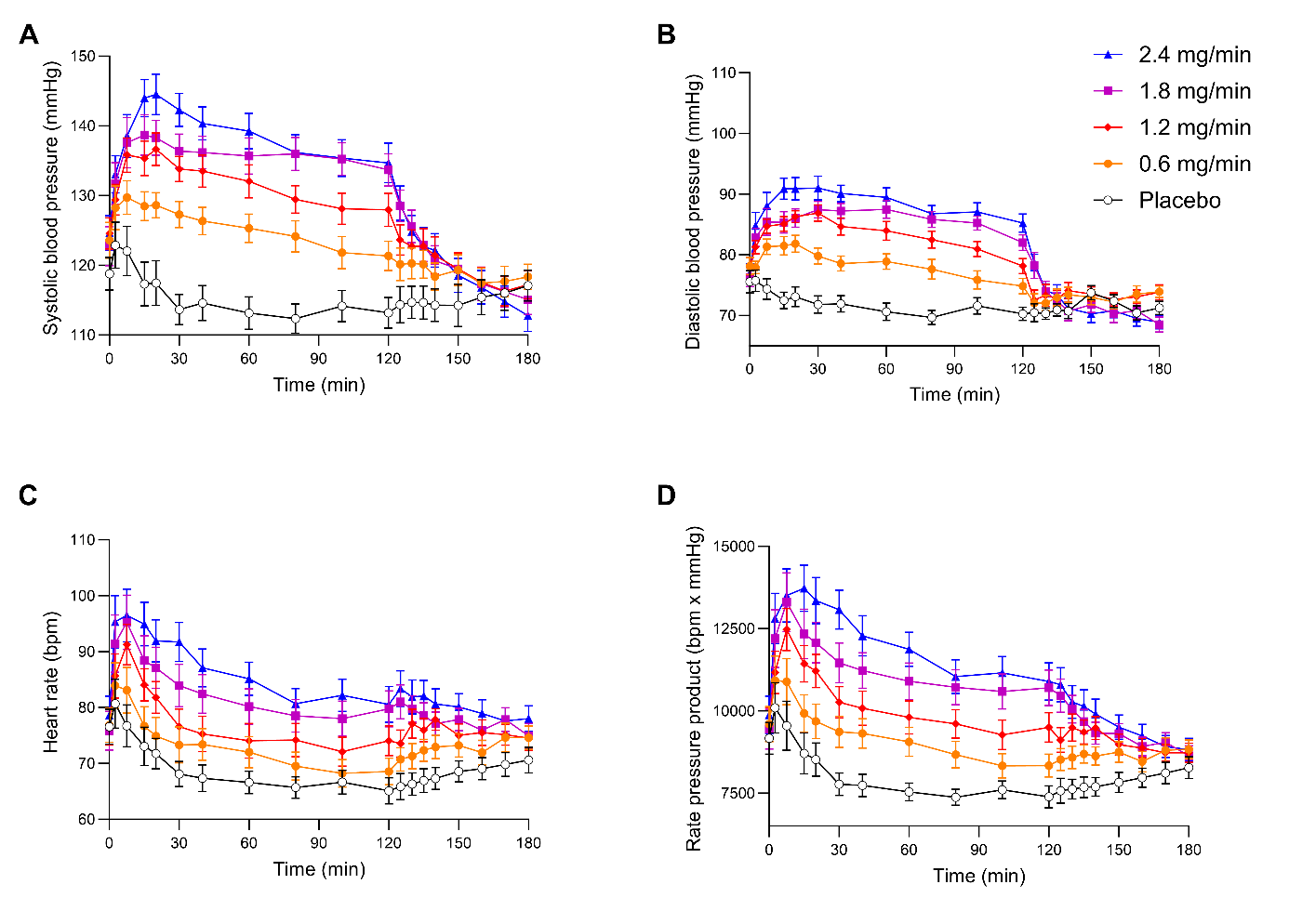


**Figure S5.** Acute autonomic effects of *N,N*-dimethyltryptamine (DMT) over time. DMT moderately and dose-dependently increased blood pressure and heart rate compared with placebo. Effects normalized within 15 min of stopping the infusion. The data are expressed as the mean ± SEM in 22 healthy participants. Maximal effects and statistics are shown in Table S1.


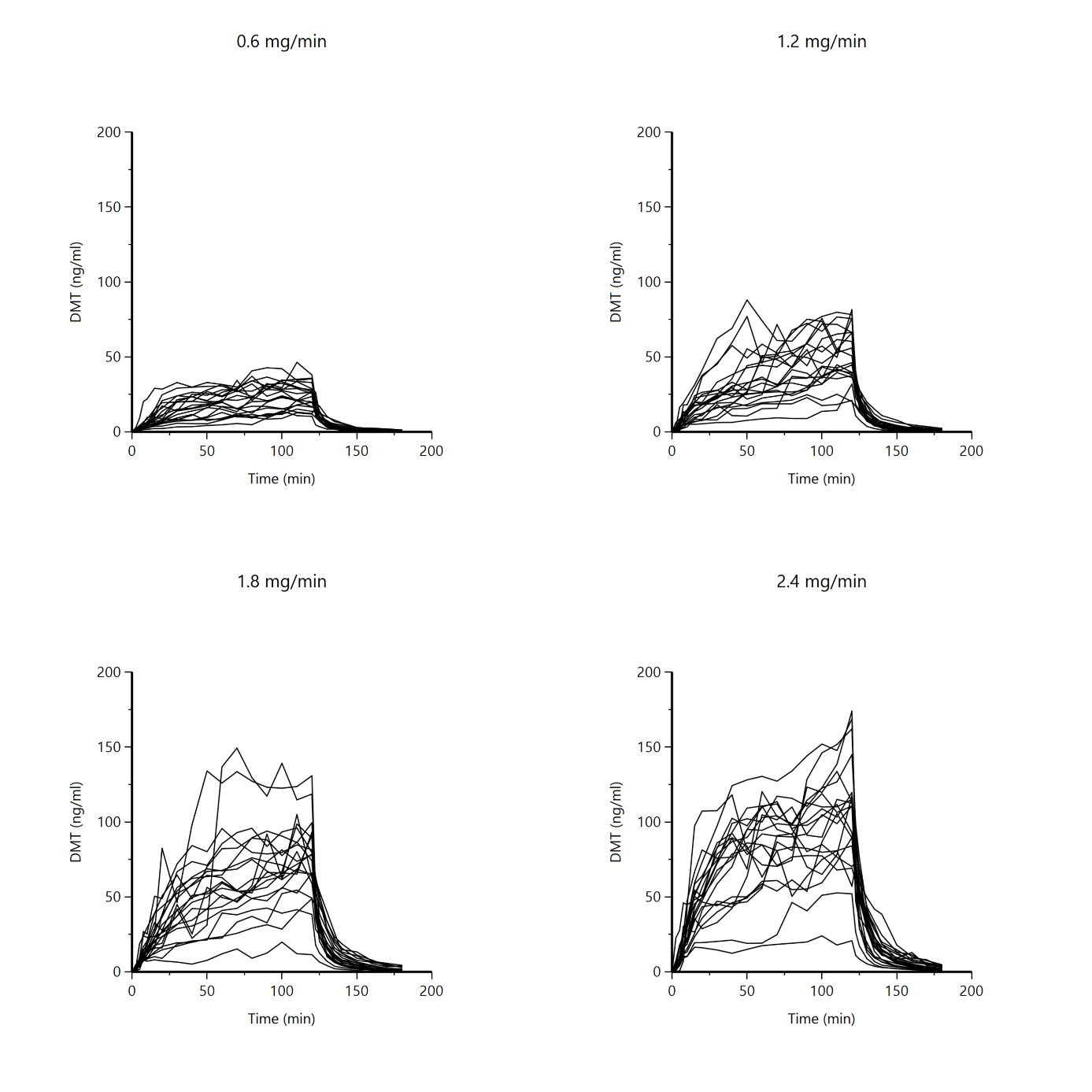


**Figure S6.** Plasma concentrations N,N-dimethyltryptamine (DMT) over time. Each line represents a single subject (*n* = 20). Infusions started at t = 0 min and lasted until t = 120 min.


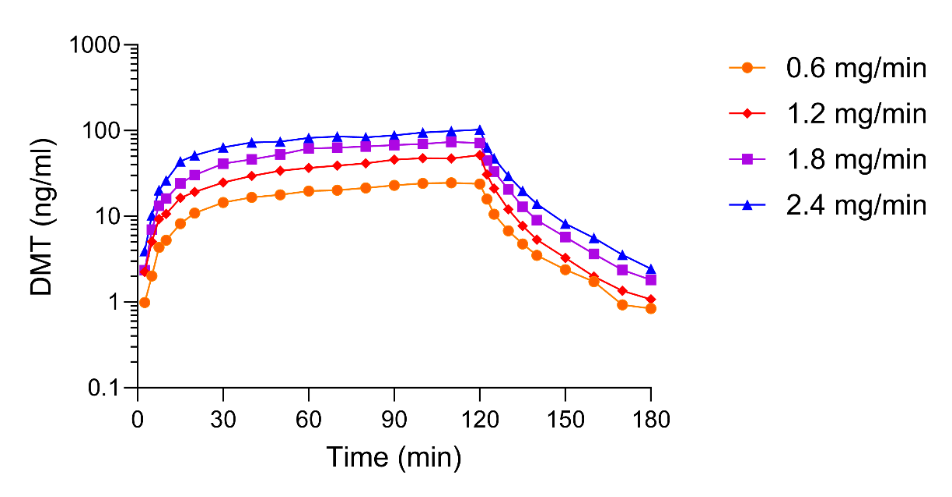


**Figure S7.** Plasma concentrations of N,N-dimethyltryptamine (DMT) over time on a semilogarithmic plot. There was an early fast elimination, with a half-life (t_1/2α_) of 6.3-7.1 min during the first 20 min after stopping the infusion, followed by a late slower decline with a half-life (t_1/2β_) of 18-19 min (Table 1, Fig. 3). The data are expressed as means in 20 healthy subjects. Error bars are not displayed because of the logarithmic scale on the y-axis.

**Table S5. CONSORT flow chart**


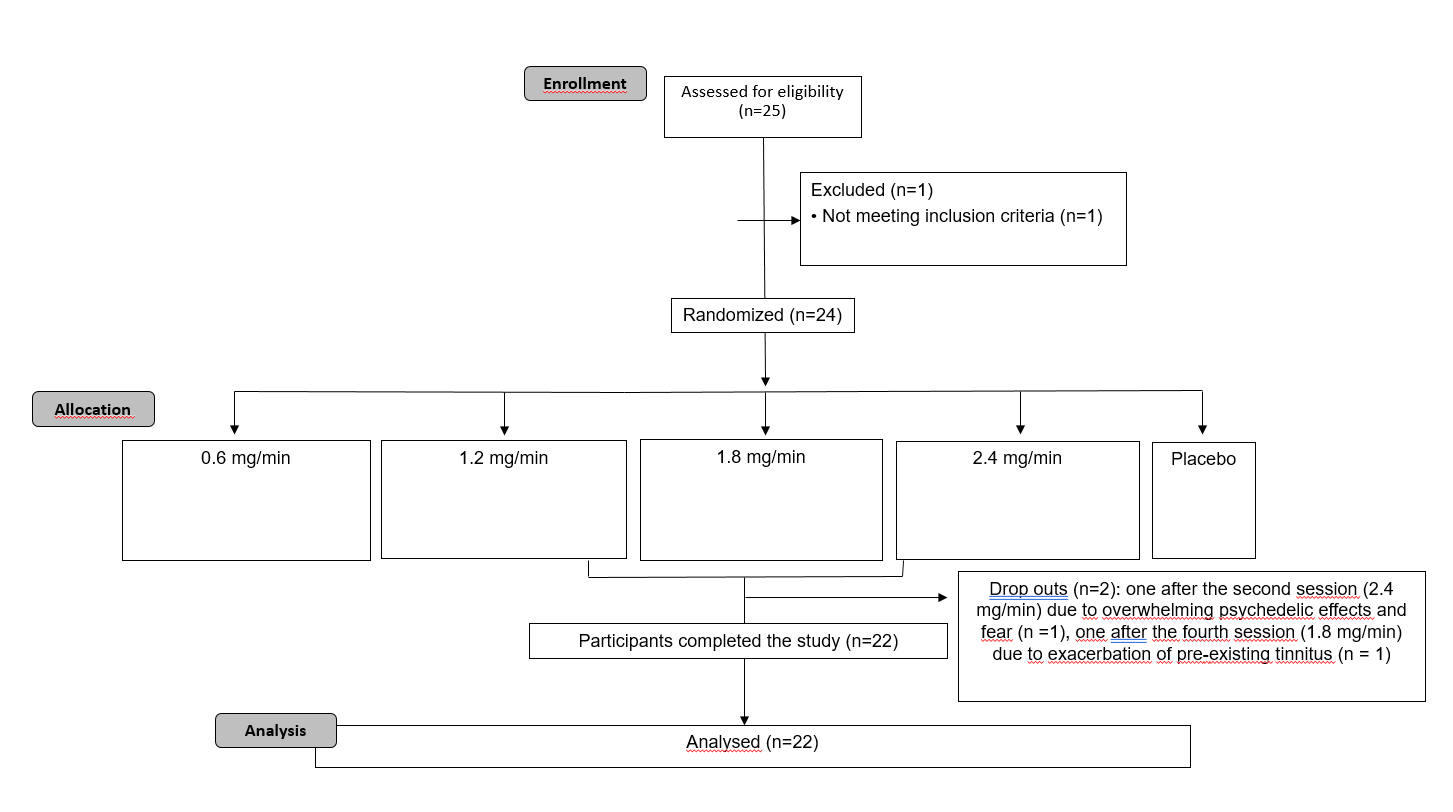


**References**

1 Riba J, McIlhenny EH, Bouso JC, Barker SA. Metabolism and urinary disposition of N,N-dimethyltryptamine after oral and smoked administration: a comparative study. Drug Test Anal. 2015;7(5):401-6.

2 Holze F, Duthaler U, Vizeli P, Muller F, Borgwardt S, Liechti ME. Pharmacokinetics and subjective effects of a novel oral LSD formulation in healthy subjects. Br J Clin Pharmacol. 2019;85:1474-83.

3 Dittrich A. The standardized psychometric assessment of altered states of consciousness (ASCs) in humans. Pharmacopsychiatry. 1998;31 (Suppl 2):80-4.

4 Studerus E, Gamma A, Vollenweider FX. Psychometric evaluation of the altered states of consciousness rating scale (OAV). PLoS One. 2010;5(8):e12412.

5 Liechti ME, Dolder PC, Schmid Y. Alterations in conciousness and mystical-type experiences after acute LSD in humans. Psychopharmacology. 2017;234:1499-510.

6 Carhart-Harris RL, Kaelen M, Bolstridge M, Williams TM, Williams LT, Underwood R, et al. The paradoxical psychological effects of lysergic acid diethylamide (LSD). Psychol Med. 2016;46:1379-90.

7 Schmid Y, Enzler F, Gasser P, Grouzmann E, Preller KH, Vollenweider FX, et al. Acute effects of lysergic acid diethylamide in healthy subjects. Biol Psychiatry. 2015;78(8):544-53.

8 Dolder PC, Schmid Y, Mueller F, Borgwardt S, Liechti ME. LSD acutely impairs fear recognition and enhances emotional empathy and sociality. Neuropsychopharmacology. 2016;41:2638-46.

9 Holze F, Vizeli P, Muller F, Ley L, Duerig R, Varghese N, et al. Distinct acute effects of LSD, MDMA, and D-amphetamine in healthy subjects. Neuropsychopharmacology. 2020;45(3):462-71.

10 Bershad AK, Schepers ST, Bremmer MP, Lee R, de Wit H. Acute subjective and behavioral effects of microdoses of lysergic acid diethylamide in healthy human volunteers. Biol Psychiatry. 2019;86(10):792-800.

11 Preller KH, Herdener M, Pokorny T, Planzer A, Kraehenmann R, Stämpfli P, et al. The fabric of meaning and subjective effects in LSD-induced states depend on serotonin 2A receptor activation Curr Biol. 2017;27:451-57.

12 Roseman L, Nutt DJ, Carhart-Harris RL. Quality of acute psychedelic experience predicts therapeutic efficacy of psilocybin for treatment-resistant depression. Front Pharmacol. 2017;8:974.

13 Griffiths RR, Johnson MW, Carducci MA, Umbricht A, Richards WA, Richards BD, et al. Psilocybin produces substantial and sustained decreases in depression and anxiety in patients with life-threatening cancer: a randomized double-blind trial. J Psychopharmacol. 2016;30(12):1181-97.

14 Stocker K, Hartmann M, Ley L, Becker AM, Holze F, Liechti ME. The revival of the psychedelic experience scale: revealing its extended-mystical, visual, and distressing experiential spectrum with LSD and psilocybin studies. J Psychopharmacol. 2024;38(1):80-100.

15 Griffiths RR, Richards WA, McCann U, Jesse R. Psilocybin can occasion mystical-type experiences having substantial and sustained personal meaning and spiritual significance. Psychopharmacology. 2006;187(3):268-83; discussion 84-92.

16 Barrett FS, Johnson MW, Griffiths RR. Validation of the revised Mystical Experience Questionnaire in experimental sessions with psilocybin. J Psychopharmacol. 2015;29(11):1182-90.

17 Becker AM, Klaiber A, Holze F, Istampoulouoglou I, Duthaler U, Varghese N, et al. Ketanserin reverses the acute response to LSD in a randomized, double-blind, placebo-controlled, crossover study in healthy participants. Int J Neuropsychopharmacol. 2023;26(2):97-106.

18 Holze F, Vizeli P, Ley L, Muller F, Dolder P, Stocker M, et al. Acute dose-dependent effects of lysergic acid diethylamide in a double-blind placebo-controlled study in healthy subjects. Neuropsychopharmacology. 2021;46(3):537-44.

19 Holze F, Ley L, Muller F, Becker AM, Straumann I, Vizeli P, et al. Direct comparison of the acute effects of lysergic acid diethylamide and psilocybin in a double-blind placebo-controlled study in healthy subjects. Neuropsychopharmacology. 2022;47(6):1180-87.

20 Straumann I, Ley L, Holze F, Becker AM, Klaiber A, Wey K, et al. Acute effects of MDMA and LSD co-administration in a double-blind placebo-controlled study in healthy participants. Neuropsychopharmacology. 2023;48(13):1840-48.
